# A DNA Regulatory Element Haplotype at Zinc Finger Genes Is Associated with Host Resilience to Small Ruminant Lentivirus in Two Sheep Populations

**DOI:** 10.3390/ani11071907

**Published:** 2021-06-26

**Authors:** Alisha T. Massa, Michelle R. Mousel, Codie J. Durfee, Maria K. Herndon, Kaneesha M. Hemmerling, J. Bret Taylor, Holly L. Neibergs, Stephen N. White

**Affiliations:** 1 Department of Veterinary Microbiology and Pathology, Washington State University, Pullman, WA 99163, USA; alisha.massa@wsu.edu (A.T.M.); mkmeyer@wsu.edu (M.K.H.); kaneesha.hemmerling@wsu.edu (K.M.H.); 2 Animal Disease Research Unit, Agricultural Research Service, United States Department of Agriculture, Pullman, WA 99163, USA; Michelle.Mousel@usda.gov (M.R.M.); Codie.Durfee@usda.gov (C.J.D.); 3 Paul G. Allen School for Global Animal Health, Washington State University, Pullman, WA 99163, USA; 4 Range Sheep Production Efficiency Research, Agricultural Research Service, United States Department of Agriculture, Dubois, ID 83423, USA; bret.taylor@usda.gov; 5 Department of Animal Science, Washington State University, Pullman, WA 99163, USA; neibergs@wsu.edu; 6 Center for Reproductive Biology, Washington State University, Pullman, WA 99163, USA

**Keywords:** small ruminant lentivirus, sheep, post-infection control, genetic fine map, validation

## Abstract

**Simple Summary:**

Sheep are affected by a viral infection that causes an incurable and difficult to treat lung, joint and brain disease that decreases production efficiency. This small ruminant lentivirus is closely related to human immunodeficiency virus (HIV) that causes AIDS in humans. Differences in breed susceptibility to disease are known in sheep that indicate genetic, or hereditary, resilience factors exist. Our objective was to study the source of this hereditary advantage so that it can eventually be translated into a tool for sheep breeders to improve herd health. Previously, one such hereditary region was detected, but little was known about possible mechanisms or nearby mutations. Here, we report several mutations that may underlie this hereditary mechanism and are in regions of DNA that are known to affect genes by increasing or decreasing gene expression, akin to gene “on/off switches.” These mutations were strongly associated with a predictor of disease severity in live animals and had a greater predicted effect on the degree of disease than previously studied mutations. Statistical association (*p* < 0.05) of disease was demonstrated in two different groups of sheep that were reared in different environments, which indicates increased likelihood that a genetic factor is producing this effect.

**Abstract:**

Small ruminant lentivirus (SRLV) causes Maedi-Visna or Ovine Progressive Pneumonia in sheep and creates insidious livestock production losses. This retrovirus is closely related to human immunodeficiency virus and currently has no vaccines or cure. Genetic marker assisted selection for sheep disease resiliency presents an attractive management solution. Previously, we identified a region containing a cluster of zinc finger genes that had association with ovine SRLV proviral concentration. Trait-association analysis validated a small insertion/deletion variant near *ZNF389* (rs397514112) in multiple sheep breeds. In the current study, 543 sheep from two distinct populations were genotyped at 34 additional variants for fine mapping of the regulatory elements within this locus. Variants were selected based on ChIP-seq annotation data from sheep alveolar macrophages that defined active cis-regulatory elements predicted to influence zinc finger gene expression. We present a haplotype block of variants within regulatory elements that have improved associations and larger effect sizes (up to 4.7-fold genotypic difference in proviral concentration) than the previously validated *ZNF389* deletion marker. Hypotheses for the underlying causal mutation or mutations are presented based on changes to in silico transcription factor binding sites. These variants offer alternative markers for selective breeding and are targets for future functional mutation assays.

## 1. Introduction

Ovine lentivirus (OvLV) exists as a collection of viral subtypes in sheep also termed small ruminant lentiviruses (SRLV) [1]. These are retroviruses which infect both sheep and goats and belong to the same genus as human immunodeficiency virus (HIV). Conserved host factors have been discovered that effect viral restriction of lentiviruses in both humans and small ruminants, such as TRIM5α [2]. The SRLV retrovirus is macrophage-tropic and incorporates into the host DNA through reverse transcription that results in pervasive life-long infection [3,4]. In sheep, the disease is referred to as Ovine Progressive Pneumonia in the U.S. and Maedi-Visna in many other countries [5], because it targets the lungs, central nervous system and joints. SRLV was documented to cause production losses by increased lamb mortality [6], reduced lamb weights in diseased older ewes [7], reduced fertility [8], and early culling of breeding stock [9]. These losses can be difficult to quantify and are frequently underestimated in individual flocks since producers may not test all animals or maintain quantitative records on all potential measures of lost production [10]. Nonetheless, economic and production efficiency consequences of SRLV are significant since 36% to 66% of commercial flocks in the U.S. contained positive sheep on serological studies [11,12,13]. Complete eradication is impractical for many flocks since retroviruses have a very small minimum infectious dose [14], and serology is the standard diagnostic testing method available with imperfect sensitivity [15,16]. Once infected there is no cure, and no approved vaccine is commercially available to prevent infection [17]. Selective breeding for sheep that better control the virus, after infection, is an option to combat economic losses and welfare issues associated with SRLV without requiring complete eradication from the flock.

Previously, a genome-wide association study identified a locus containing zinc finger genes that were associated with host control of the virus and lesion severity after infection [18,19]. Proviral load in the blood has been documented to correlate with histological lesion severity in sheep [19] and is likely a useful phenotype to predict disease resiliency in sheep since this is well documented in humans with HIV [20]. Early efficient control of viral replication is generally associated with reduced disease severity for HIV and other lentiviruses [21]. Proviral load in humans is associated with disease severity, clinical progression, treatment response and is documented to be very low to exceptionally low in slow progressors and elite controllers infected with HIV [20,21,22,23,24]. In sheep, we are defining the phenotype of reduced proviral load as resiliency to small ruminant lentivirus because it is a marker of control of the pathogen after infection potentially involving aspects of disease tolerance and is not considered resistance, which is defined as imperviousness to infection or elimination of infection [25]. Breeding for tolerance or resiliency may create less selective pressure on the pathogen than breeding for strict resistance [26]. The locus contains four zinc finger genes *ZNF389*, *ZKSCAN8*, *ZSCAN16,* and *ZNF165* [27,28,29] that had not been otherwise documented to affect viral infections in vivo. However, other zinc finger proteins, such as ZAP, have been shown to restrict retroviruses [30]. In fact, this innate host factor has been shown to restrict viral replication in a variety of retroviruses from several species [1]. Zinc finger genes have also been demonstrated to coevolve in the host with endogenous and exogenous retroviruses [31,32]. Eight markers across the locus, mostly within genes, were tested in previous work, and a single insertion/deletion marker (rs397514112) near *ZNF389* was validated in three sheep populations [33]. This marker and the haplotype it tracks were found to have no negative associations with production traits [34], meaning this locus could be used for selective breeding without obvious unintended negative effects.

The four zinc finger genes in the region associated with lentiviral resiliency in sheep are the Cys2/His2-type (C2H2) and include Kruppel associated box (KRAB) domains with known repressive function [35]. C2H2 zinc finger genes contain repeated domains that form finger-like protein structures of 28–30 amino acids which bind the DNA helix. C2H2 zinc finger proteins are one of the largest families of transcription factors in eukaryotic genomes, however those with greater than three zinc finger domains at the C-terminus such as *ZNF389*, *ZKSCAN8*, *ZSCAN16* and *ZNF165*, are the most poorly understood [36], and prediction of their DNA binding sequence becomes less accurate when there are higher numbers of zinc finger domains. Domestic cattle and pigs, other artiodactyl species related to sheep, have annotated orthologs of *ZNF389*; *ZKSCAN8*, *ZSCAN16,* and *ZNF165* are annotated in many mammalian species including humans in the current Refseq annotations [37,38,39,40]. In humans, a pseudogene *ZKSCAN8P1*, has been implicated as *ZNF389* but it is currently unclear if this is an orthologous gene to *ZNF389* annotated in sheep. At the time of the association analysis of the zinc finger gene region in sheep [33], the specific functional implications for the intergenic validated marker near *ZNF389* and the zinc finger genes were unknown.

Reported here, further assessment of polymorphisms in the region were completed to identify the functional importance of these variants and to examine additional markers that may be useful for genetic marker-assisted selection. DNA regulatory elements were previously annotated by chromatin immunoprecipitation and high throughput sequencing (ChIP-seq) for histone post-translational modification marks including H3K27ac and H3K4me3 in sheep [41] and used to focus the search for polymorphic variants of interest to the SRLV resilient phenotype. These regulatory elements are associated with reproducible biological functions such as gene enhancers and promoters. For instance, the histone modification H3K27ac denoted active chromatin regions typically associated with enhancers [42,43]. Chromatin that has the histone modification H3K4me3 and H3K27ac near the transcription start site of a gene defines active promoter elements that indicate active gene expression [44,45].

Associations were tested across 34 variants within the eight regulatory elements recently annotated [41] in this chromatin domain. A haplotype within experimentally determined active DNA regulatory elements from previously determined ChIP-seq data in sheep was associated with a resilient phenotype against small ruminant lentivirus. Variants within active cis-regulatory elements such as promoters and enhancers often have functional consequences for transcription factor binding and gene expression which will be explored further. These results are consistent with the model that most causal mutations of complex disease phenotypes are within noncoding regulatory elements rather than coding regions of genes and influence gene expression [46,47,48].

## 2. Materials and Methods

### 2.1. Ethics Statement

Animals were cared for and handled according to protocols approved by the Washington State University Institutional Animal Care and Use Committee (ASAF 4618 and 6632) or by the U.S. Sheep Experiment Station Institutional Animal Care and Use Committee (protocols: 10-06 and 10-07).

### 2.2. DNA Extractions and Genotyping

Whole blood was collected via jugular venipuncture into EDTA-vacutainer tubes for DNA extraction as previously described [31] from two sheep populations in a total of 543 animals. Rambouillet purebred ewes (population 1) were sampled at the U.S. Sheep Experiment Station in Idaho and crossbred (Rambouillet, Columbia) ewes (population 2) from a privately owned production flock in Montana. Population 1 consisted of 164 small ruminant lentivirus infected ewes between one to five years of age. Flock SRLV prevalence for population 1 was 42.2% [33]. Population 2 consisted of 379 ewes, between one to six years of age, selected by a random number generator from the 533 SRLV infected animals in the flock. Older infected animals aged seven to eight years were excluded for estimability due to low numbers of animals in these groups since age was included in the model. Population 2 SRLV prevalence was 87.2%. Additional characteristics of the populations were reported in previous publications [18,33]. No animals were shared between population groups. The GeneCatcher gDNA Blood Kit (Invitrogen, Life Technologies, Carlsbad, CA) was used according to manufacturer’s instructions for DNA extraction.

Four individual Rambouillet animals were selected from population 1 based on alternate homozygous genotype status at the *ZNF389* validated marker for whole genome high-throughput sequencing (Rambouillet) in order to select variants within regulatory elements for further genotyping in all 543 sheep. These four Rambouillet were selected to fit the overall population trend, in that the two insertion homozygous animals had low proviral concentrations and two deletion homozygous animals had high proviral concentrations. Two additional uninfected Rambouillet from the same flock as population 1 and two crossbred sheep (Suffolk, Polypay, Rambouillet, Targhee) from a Washington research flock [41], were sequenced at high depth and included in evaluation for DNA variants to screen for rare alleles (crossbred sheep). Paired-end sequencing (Illumina) produced between 600–700 million reads per animal that passed read quality and adapter trimming with BBDuk from the BBTools suite (Bushnell, 2021, http://sourceforge.net/projects/bbmap/) (accessed on 1 November 2020). Reads were aligned to the Rambouillet reference genome (Oar_Rambouillet_v1.0, GCF_002742125.1) [27] with BWA [49]. Aligned reads were quality filtered, sorted, and indexed with SAMtools [50].

The region on chromosome 20 between 32.92 Mb and 33.01 Mb was evaluated for variants in the sequenced Rambouillet sheep by comparison to the Rambouillet reference genome and the sequenced crossbred sheep. Variants were selected by sequence inspection of aligned reads in IGV [51] that appeared to be on the same or similar haplotypes as the validated marker and were within the active regulatory elements as determined by ChIP-seq. ChIP-seq annotations in the zinc finger region were utilized from those previously published [41] and with attention to regions annotated as transcription start sites from cap analysis gene expression (CAGE) data [27]. ChIP-seq signal consensus BED files combined from two animals for the histone modifications H3K4me3 (promoters) and H3K27ac (active enhancers and promoters) and raw ChIP-seq signal BigWig tracks in each individual animal were used for regulatory element annotation. CAGE data that shows transcription start sites for genes from publicly available annotation data was overlaid with the active *cis*-regulatory elements of interest [27,41]. ChIP-seq BigWig signal tracks for CTCF (insulators) and H3K27me3 (silencers/heterochromatin) enrichment were used to define the boundaries of the chromatin domain and limit which regulatory elements and genes around the previously validated marker were included in assessment.

Taqman Genotyping Assays were performed according to manufacturer’s instructions (Applied Biosystems, Foster City, CA USA) utilizing 20 ng of genomic DNA in 10 microliter reactions for all 543 animals in both populations. Dried genomic DNA samples were incubated in liquid Polymerase Chain Reaction (PCR) reagents for 15 min at 37 °C immediately prior to PCR cycling. The PCR thermal cycler program was polymerase activation at 95 °C for 10 min, then repeating 50 cycles of 95 °C denaturing for 15 s and 60 °C annealing/extension for 1 min. Primers and probes were designed for variants by the manufacturer’s website tool https://www.thermofisher.com/order/custom-genomic-products/tools/genotyping/ (accessed on 1 November 2020). Assay identification codes and sequences for primers and fluorescent probes are provided in supplementary Table S1. Automated design for the initially selected variants failed at 11% of the sites, mostly due to a high density of additional variants nearby. Genotyping was completed at 36 variants with successful genotyping assay cluster differentiation.

### 2.3. Small Ruminant Lentivirus Phenotypes

Proviral concentration was assessed on all animals in triplicate qPCR assays as previously described [16] utilizing 1 ug of DNA extracted from peripheral blood. Only animals infected with SRLV were included in the downstream association analyses. The two populations of sheep were chosen for high SRLV prevalence within the flocks as previously described [33].

### 2.4. Statistical Analysis

Association analyses were completed in JMP Genomics version 7.1 (SAS Institute, Cary, NC USA). A standard least squares model was employed with independent variables set as genotype and age in years, and sire treated as a random effect. The dependent variable was log_10_-transformed SRLV proviral concentration. Age in years was treated as a categorical (nominal) variable to account for non-linearity of proviral concentration as sheep age. *p*-values less than 0.05 were considered significant. SRLV phenotypes were reverse-transformed (10^x^) to viral copies/ug DNA scale for indicated results. Genotypes were assessed for Hardy-Weinberg Equilibrium (HWE) with chi-squared analysis (*p* < 0.05) which yielded 34 variants for association testing. HWE *p*-values are displayed for each variant in supplementary Table S2.

### 2.5. Linkage Disequilibrium and Haplotype Analyses

Linkage disequilibrium measures were derived using Haploview v4.2 [52] r^2^ and D’ values. Haplotypes with less than 1% allele frequency were considered rare and removed from further analysis. PHASE v2.1.1 [53,54] was used to estimate population frequency of haplotypes, and genotypes were assigned for individual animals based on calculated haplotypes. Animals with haplotype assignment probabilities of less than 95% were dropped from haplotype association analysis. A standard least squares model as described above was used to evaluate haplotype association with SRLV phenotypes.

### 2.6. Transcription Factor Binding Analysis

The MATCH tool [55] was used with the TRANSFAC database (version 2021.1, geneXplain GmbH, Qiagen, Germantown, MD) of binding motif sequences to predict allelic difference in binding affinity around each selected variant. Settings for motif scanning included immune cell specific with a core and matrix match above 0.9, or vertebrate non-redundant with minimize false positives and minimize the sum of false negative and false positive results. Any transcription factor binding motif that was predicted to be created or abolished between the two alleles was recorded.

## 3. Results

### 3.1. ZNF Gene Region in Detail

The chromatin domain around the previously validated marker, rs397514112 [25], was delineated by evaluation of boundary elements from ChIP-seq for in the relevant cell type of alveolar macrophages. Evaluation of both CTCF insulators and H3K27me3 silencers delimited the domain as extending from approximately 32.92 Mb to 33.01 Mb on Rambouillet_v1.0 chromosome 20. These boundaries were affirmed by the presence of annotated tRNA genes.

**Figure 1:**
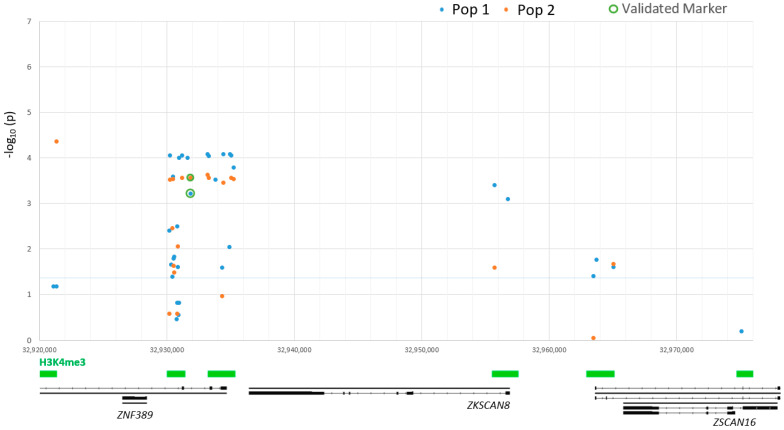
Association with small ruminant lentivirus proviral concentration at variants within regulatory elements for zinc finger genes on ovine chromosome 20. ChIP-seq annotations for H3K4me3 enrichment are shown as green bars at the bottom, above Refseq gene annotations in black. Population 1 (purebred Rambouillet) are identified by blue dots and population 2 (crossbred Rambouillet, Columbia) are identified by orange dots. The previously validated marker (rs397514112) is highlighted by green circles for each population.

Four annotated genes were found within the region, *ZNF389* (NCBI Gene ID 101104612), *ZKSCAN8* (NCBI Gene ID 101121465), *ZSCAN16* (NCBI Gene ID 101104351) and *ZNF165* (NCBI Gene ID 106990266), each containing C2H2 zinc finger repeats at the C-terminus and KRAB or SCAN domains near the N-terminus. Each gene had a proximal 5′ active *cis*-regulatory element defined by both H3K4me3 and H3K27ac enrichment of the genome. *ZNF389* also had two downstream, within-gene, smaller regulatory elements. CAGE data indicated there were three transcription start sites for *ZNF389*, one within each of the ChIP-seq regulatory elements. There were two additional active, proximal regulatory elements upstream of two predicted lncRNAs, *ZSCAN16-AS1* (NCBI Gene ID 105603808) and LOC114108620 (NCBI Gene ID 114108620). The regions searched for variants included approximately 14,500 bp of DNA in the eight total active regulatory elements. Taqman genotyping assays were performed and analyzed for 34 variants including 30 single nucleotide polymorphisms (SNP), one multiple nucleotide polymorphism (MNP) and three small insertion/deletion variants that varied from 1–3 bp. Greater than 95% of animals were successfully genotyped, and all variants passed HWE analysis (*p* > 0.05), see supplementary Table S2 for chi-squared results for population 1 and supplementary Table S3 for population 2. In addition, the previously validated insertion/deletion marker (rs397514112) was included for reference. Twenty-four of the variants were within the two regulatory elements that immediately flanked the previously validated marker (Figure 1). The remainder of the variants were distributed amongst five additional regulatory elements within 57.5 Kb in the 3′ direction and within 10.7 Kb in the 5′ direction from the validated marker.

### 3.2. Population One Association Analysis

Within population one, 164 SRLV positive animals were included in the association analyses. Overall, twenty-eight of the variants had significant association with SRLV proviral concentration (*p* < 0.05) shown in Figure 1. Variant minor allele frequencies ranged from 0.48 to 0.02 in purebred Rambouillet sheep (Table 1). For all variants with a minor allele frequency greater than 0.05, the heterozygous animals adjusted mean proviral concentration was not significantly different from the resilient homozygote adjusted mean.

At the previously validated marker, the association with SRLV remained (*p* = 6.00 × 10^−4^) with a proviral concentration log-transformed difference of 0.803 (Table 2) between animals with a homozygous deletion and those with a homozygous insertion. This corresponded to a reverse-transformed adjusted mean of 24.4 viral copies/ug of DNA in the insertion homozygotes and 155.2 viral copies/ug of DNA in the more susceptible deletion homozygotes. Additionally, 14 variants tested had improved *p*-values (*p* = 4.00 × 10^−4^ to *p* = 5.08 × 10^−7^) and greater effect sizes as calculated by the log_10_ proviral concentration difference between homozygous genotypes when compared to the previous validated marker as shown in Table 2. Variants within three separate regulatory elements for *ZNF389* and *ZNF192* (*ZKSCAN8*) had the highest log-transformed adjusted mean proviral concentration difference between genotypes of 0.838 to 0.967 in population 1. The reverse-transformed adjusted mean proviral concentrations were 32.7 viral copies/ug DNA and 303.4 viral copies/ug DNA for alternate homozygous genotypes at the most extreme variant (rs420471584) which was 24 Kb 3′ from the previous validated marker.

### 3.3. Population Two Association Analysis

Population two was genotyped at 20 variants which had association with SRLV resiliency in population one. In total, 378 animals were retained in analyses as one animal was removed for high genotyping failure rate (>10% of variants tested). Minor allele frequency in the crossbred population ranged from 0.47 to 0.04 (Table 1). Fifteen variants across five different regulatory elements were associated with SRLV proviral concentration (*p* < 0.05), see Table 2. Eight of the variants in the haplotype had a larger adjusted mean log-transformed proviral concentration difference between homozygous genotypes than the previous validated marker, and nine of the variants had greater significance (lower *p*-values), as displayed in Table 2. For nearly all variants tested in this population, the adjusted mean proviral concentration in heterozygous animals was not different from the susceptible homozygote (*p* > 0.05).

### 3.4. Significant Variants in Multiple Populations Were in Two Regulatory Elements

**Figure 2:**
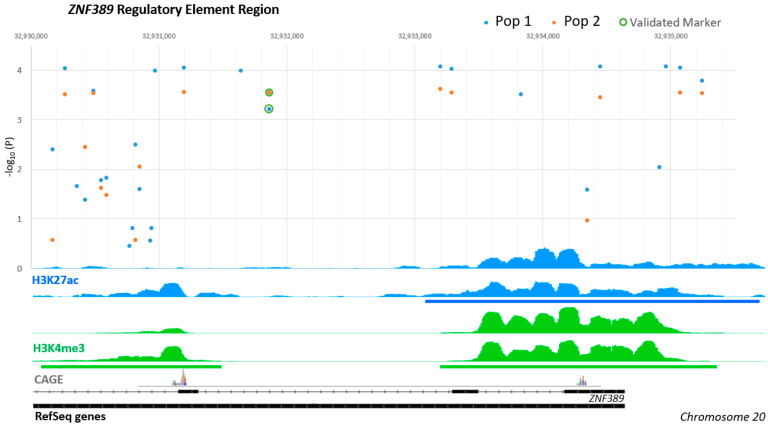
Association with SRLV proviral concentration at higher resolution for variants within the two regulatory elements near ZNF389 that flank the previously validated insertion/deletion marker (rs397514112) on chromosome 20. Below, the ChIP-seq signal tracks for two animals enriched for H3K27ac (active proximal enhancers) and H3K4me3 (active promoters) is displayed. Transcription start site data from cap analysis gene expression (CAGE) from alveolar macrophages is also displayed [27]. Peak calls significant in both animals are indicated by the solid blue or green bars. Refseq gene annotation is displayed in black along the bottom.

In both populations, the most significant and extreme variants were located within the two regulatory elements that flank the *ZNF389* insertion/deletion marker (Figure 2). These two active regulatory elements were located at 32.931 Mb and 32.935 Mb on chromosome 20. The larger element was approximately 2100 base pairs in length and the smaller downstream element was 1400 bp in length. The larger regulatory region was predicted to contain approximately 29 total variants within Rambouillet, including rare variants and those for which Taqman allelic discrimination assays could not be designed. The smaller regulatory element was predicted to contain 17 total variants within Rambouillet. The alleles across the markers tested in these elements are displayed in Table 2 according to association with resiliency or susceptibility to SRLV. The same alleles were associated with resiliency and susceptibility in both populations.

### 3.5. Haplotypes and Phenotypic Association with Common Haplotypes

**Figure 3:**
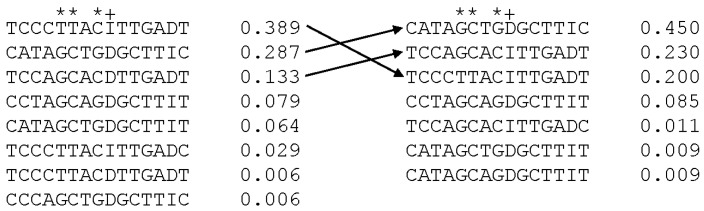
Haplotypes and population frequency as determined by PHASE. On the left are the haplotypes within population 1 (purebred Rambouillet) and on the right are haplotypes for population 2 (crossbred, Rambouillet, Columbia). The same three haplotypes were the most common in both populations. The fifteen loci are shown in columns listed in the same order as in Table 1 and Table 2, with the previously validated marker listed as +. An asterisk (*) is above each locus in the two key regulatory elements upstream of *ZNF389*. For loci 09 and 14, D represents the deletion allele and I represents the insertion allele.

Linkage disequilibrium analysis showed that 7 of the 15 significant markers were in exceptionally strong LD with the previously validated marker (r^2^ > 0.95) in population 1 (see Table 2) and 8 of the 15 significant markers were in exceptionally strong LD with the previously validated marker in population 2. Haplotypes were assessed across the 15 significant markers in each population and yielded three common haplotypes that were present for both animal groups in greater than 10% of the population (Figure 3). Three shortened common haplotypes were determined with PHASE analysis that consisted of the markers in the two key regulatory elements: rs414155747, rs425583788, rs407355422 and rs397514112 (validated marker). Haplotype 1 had alleles GCGD (D = deletion, A and I = insertion, AAT) across these four markers and was most common in both populations, with a frequency of 0.436 in population 1 and 0.554 in population 2. Haplotype 2 had alleles TTCI with a frequency of 0.427 in population 1 and 0.201 in population 2. Haplotype 3 was composed of the alleles GCCI with a frequency of 0.125 in population 1 and 0.241 in population 2. Haplotype 1 was highly associated with an SRLV susceptible phenotype in population 1 (*p* = 2.72 × 10^−5^) in a dose dependent manner (0, 1 or 2 copies of the haplotype) and in population 2 (*p* = 5.82 × 10^−4^). The reverse transformed adjusted mean proviral concentration for population 1 was 226 viral copies/µg DNA for two copies of the haplotype, 49.8 viral copies/µg DNA for one copy of the haplotype and 29.4 viral copies/µg DNA for zero copies of haplotype 1. Haplotype 2 was also associated with SRLV proviral load, but was protective with one or two copies of the haplotype associated with a similar resiliency effect (*p* = 2.10 × 10^−3^ for population 1 and *p* = 2.94 × 10^−2^ for population 2). Haplotype 3 was not significantly associated with SRLV phenotype (*p* = 0.09 and *p* = 0.11 for populations 1 and 2).

### 3.6. TRANSFAC Predictions

The variants associated with SRLV proviral concentration that were validated in both populations were analyzed using the TRANSFAC database to identify transcription factor binding motifs. Ten of the variants including a single insertion/deletion variant and a single MNP (2 bp) had predicted differences in transcription factor binding based on the allelic sequence. All variants, except one, were within the two *ZNF389* regulatory elements that flank the previous validated marker. Transcription factor proteins implicated in unique binding at one of the alleles included GATA3, c-JUN, TCF-1, LEF-1, Ikaros, ZNF333, AP-2alpha, NFATc2 and HNF-3beta. Ubiquitous proteins such as AP-1 and SP100 were also implicated in predicted binding differences. At the rs427575002 variant, the repressive transcription factors BEN and Kaiso had predicted differences in binding affinity. The variant that is predicted to bind these repressors was found within the smaller downstream *ZNF389* regulatory element which had an annotated difference in ChIP-seq signal in the region between alternate homozygote animals (see Figure 2).

## 4. Discussion

The zinc finger chromatin domain examined in sheep represents a synteny block conserved from humans to artiodactyls. However, one major difference in humans compared to sheep is that *ZNF389* is annotated as a pseudogene paralog of *ZKSCAN8* (*ZNF192*) in humans. *ZNF389* is an interesting gene target for a novel viral restriction factor as it may have functionally diverged in the caprine subfamily in response to the recent emergence of small ruminant lentiviruses [29,32,56]. Maedi-visna, ovine progressive pneumonia and caprine arthritis and encephalitis are caused by multiple subtypes of the same slow viral disease in sheep and goats that have emerged in the last century [57] and cause multisystemic wasting characterized by chronic lymphocytic pneumonia, arthritis, mastitis, and encephalitis. The poly-zinc finger transcription factor gene family consists of over 700 members and examination across host clades indicates that these genes arose from repeated duplication events followed by functional divergence [32,58]. Generally, positive selection for new transcriptional repression activity in response to transposable retroelements and retroviruses has been found or hypothesized [59,60]. Zinc finger transcription factors also have emerging roles in modulating the innate immune response [61] and therefore viral restriction factors in one species may be protective against retroviruses from another species. There are other well-known examples of host viral restriction factors that affect retroviruses from multiple species, such as APOBEC3, Tetherin, ZAP and TRIM5α [30,56,62].

Through fine mapping of this region on ovine chromosome 20, we identified several candidates for selection markers which may prove more useful for predictive breeding decisions in other breeds of sheep than the single previously validated marker. This region appears to contain a haplotype block of variants that are significantly associated with SRLV resiliency within two active regulatory elements upstream of the *ZNF389* gene. This could indicate that these regulatory elements are functionally linked and influence gene expression together or that more than one sequence variant may be playing a causal role in the phenotype. These variants validated in two populations of sheep from different breed backgrounds and under different environmental conditions and had greater significance than the previously validated marker. Studies have also highlighted variability in utility of a single genetic marker for trait selection in different breeds or environments [5], thus it is important to mention that the related breed composition of the two populations in this study is a limitation to broader assumptions on SRLV association with these markers. In particular, the *TMEM154* mutations for odds of infection have been shown to associate with reduced risk [63,64] in American and European sheep and with improved control post-infection in some U.S. sheep [65]. However, *TMEM154* mutations were not associated with SRLV infection in different breeds in several populations [66].

Importantly, the effect size is greater at some of these markers (MNP: rs414155747 and rs425583788, rs407355422) compared to the previously validated marker (rs397514112). We hypothesize that these variants would cause a change in expression of the zinc finger genes that restrict viral replication. The discovery of this haplotype within the regulatory elements of zinc finger genes lends further support that one or multiple host zinc finger genes may function as a viral restriction factor in sheep.

Genome-wide association studies in humans and livestock indicate that the majority of functional variants lie within DNA regulatory elements [46,67,68,69]. Annotation of these regulatory elements in relevant cell- and tissue- types will be of increasing importance to elucidate the biology behind functional mutations in food producing species. Published alveolar macrophages ChIP-seq data [41] was leveraged to evaluate active regulatory elements in the chromosome region and identify the boundaries of the putative chromatin domain since these cells are the primary phagocyte of the lungs. The distance searched for variants was also limited based on decline of linkage disequilibrium with distance in the same population of sheep (r^2^ < 0.25 at 35 Kb) [18]. Out of eight total regulatory elements evaluated within the region, the most significant variants were present within two adjacent *cis*-regulatory elements nearest to *ZNF389* flanking the previously validated marker (Figure 1 and Figure 2).

Determination of regulatory elements with trait associations is important because polymorphisms have been detected in intergenic regions associated with critical economic traits in livestock such as polledness [70]. The vast wealth of human literature indicates that it is most common for genome-wide association studies to find significant polymorphisms for traits within promoters and enhancers rather than within coding regions of genes [46,71]. Breaking research within livestock species indicates a similar trend emphasizing the importance of studying these regulatory elements in many species [69]. *Cis*-acting DNA regulatory elements control gene expression by serving as structural elements for many *trans*-acting proteins to promote or disrupt gene expression [72]. Promoters marked by H3K4me3 are often present at many genes in the cell type that are not actively expressed [41,44]. However, if the region is also marked at nucleosomes by H3K27ac, this is highly associated with gene expression. The zinc finger gene regulatory elements identified to contain the haplotype cluster had enrichment for both H3K27ac and H3K4me3 in macrophages indicating they may be expressed in this key cell type for lentiviral infection. Examination of CAGE data [27] showed that each of the two *ZNF389* regulatory elements had a narrow region of active mRNA transcription. Other members of this zinc finger family have been implicated as transcriptional repressors; we hypothesize that increased expression of one of these genes due to promoter variants may cause increased transcriptional repression to the viral genome or provirus. ChIP-seq data in sheep indicate potential insulators enriched by CTCF around *ZNF389* and *ZNF192* which would pose the most likely gene effectors. However, these four, and many other zinc finger genes on the same chromosome have been linked to similarly located active *cis*-regulatory elements in humans from the ENCODE cell line data [73].

Evaluation of predicted in silico transcription factor binding motifs between alleles for each variant resulted in discovery of putative functional differences in these two active regulatory elements. This method may reveal functional variants that cause changes in transcription factor protein binding [74,75], as described here, and found in livestock at gene promoters for other traits of economic significance [48]. Analysis of transcription factor binding changes yielded hypotheses for function of ten of the associated variants from within the two *ZNF389* regulatory elements that were validated in both sheep populations. Several of the transcription factors with predicted changes have been implicated to various degrees in altering the host response to viruses or altering viral replication. Some of these transcription factors have been directly shown to interact with viruses such as LEF-1 in the case of HIV [76]. However, binding of transcription factors to host promoters would be an indirect effect. We hypothesize transcription factors function through expression alteration in zinc finger genes which then mediate a downstream direct effect on the virus. In mice, Gata3 has been implicated in altered anti-viral immunity [77], which was one of the transcription factors we predicted to have differential binding affinity to sheep promoters in the region. Functional testing for molecular effects of these variants were outside the scope of this research study but will be performed in the future such as electrophoretic mobility shift assays and promoter reporter assays.

Regulation of gene expression appears to be complex within this region. For example, there is an active regulatory element for both *ZSCAN16* and *ZSCAN16-AS1* which is an antisense lncRNA. Antisense RNA can act as a sponge for miRNA and protect the complementary mRNA transcript from the effects of miRNA, creating several layers of ncRNA regulation for a single gene. However, this study cannot rule out long distance interactions of these regulatory elements or genes with distant *cis*-regulatory elements along the chromosome. Chromatin folding has occasionally been implicated in functional association of gene-enhancer loops at distances of up to 1 Mb. Additionally, since several of these variants are in high linkage disequilibrium with the previously validated marker, this study does not fully resolve linkage association from biological function in the tested populations.

## 5. Conclusions

Overall, a haplotype of variants within two active *cis*-regulatory elements for *ZNF389* was identified that improved the association and/or effect size for small ruminant lentivirus proviral concentration as a live-animal measure of lesion severity in Rambouillet and related crossbreeds from the Western U.S. This provision of additional variants will prove useful in selective breeding decisions in the future, although the utility of phenotype predictive value in sheep from different breeds or geographical locations remains unknown. Ten variants were identified that had predicted transcription factor binding differences between alleles. One or more of these variants can be incorporated into a commercial test to combat SRLV infection which ultimately has potential to improve efficiency of meat, milk, and wool production. Since several viral restriction factors previously known affect lentiviruses from multiple species, a novel viral restriction factor identified in sheep may have implications for interactions between host and retroviruses in additional species.

## Figures and Tables

**Table 1:** Minor allele frequency at each variant in the sheep population 1 purebred Rambouillet and population 2 crossbred (Rambouillet, Columbia) sheep. Allele nucleotide bases are shown for SNPs: adenine (A), cytosine (C), guanine (G), and thymine (T), I = insertion and D = deletion for small indels. Total animals genotyped for each population (*n*). See supplementary Table S2 for animal counts by genotype. * Previously validated marker.

Marker	Population 1 *n* = 164 Minor Allele Freq		Population 2 *n* = 379 Minor Allele Freq	
rs193645606	C	0.44	T	0.45
rs418789060	A	0.35	A	0.47
rs161334222	T	0.44	C	0.44
rs398476053	C	0.42	C	0.20
rs414155747&rs425583788	TT	0.43	TT	0.20
rs427575002	T	0.36	T	0.47
rs407355422	G	0.44	C	0.44
rs397514112 *	D	0.44	I	0.44
rs599110985	G	0.44	T	0.44
rs411076283	C	0.44	T	0.44
rs407841455	T	0.44	G	0.44
rs161334287	T	0.44	A	0.44
rs598937573	I	0.44	D	0.44
rs406431156	C	0.33	C	0.47

**Table 2:** Genotypes at each regulatory element marker tested that yielded a significant association (*p* < 0.05) with SRLV phenotype of resilience in sheep. The most extreme genotypes associated with resiliency and susceptibility to SRLV and log-transformed adjusted mean proviral concentration difference between genotypes (Genotypic log10 Conc. Diff.) for Population 1 (purebred Rambouillet) is shown. The *p*-value calculated for the association analysis in population 1 and linkage disequilibrium (r^2^) between each newly tested marker and the previously validated marker for both population 1 and population 2 is also shown. Genotype allele nucleotide bases are shown for SNPs: adenine (A), cytosine (C), guanine (G), and thymine (T), I = insertion and D = deletion for small indels. See supplementary Table S4 for additional data on population 2. * Previously validated marker.

Marker	Resilient Genotype	Susceptible Genotype	Genotypic Log_10_ Conc. Diff.	*p*-Value	LD * Pop. 1 (r^2^)	LD * Pop. 2 (r^2^)
rs193645606	T/T	C/C	0.857	8.95 × 10^−5^	0.950	0.989
rs418789060	C/C	A/A	0.537	4.06 × 10^−2^	0.664	0.705
rs161334222	C/C	T/T	0.830	2.57 × 10^−4^	0.926	0.989
rs398476053	C/C	A/A	0.434	1.64 × 10^−2^	0.537	0.312
rs414155747&rs425583788	TT/TT	GC/GC	0.445	1.46 × 10^−2^	0.556	0.313
rs427575002	A/A	T/T	0.572	2.46 × 10^−2^	0.690	0.678
rs407355422	C/C	G/G	0.859	8.70 × 10^−5^	0.950	0.989
rs397514112 *	I/I ^1^	D/D	0.803	6.00 × 10^−4^	-	-
rs599110985	T/T	G/G	0.857	8.39 × 10^−5^	0.950	0.989
rs411076283	T/T	C/C	0.856	9.20 × 10^−5^	0.950	0.989
rs407841455	G/G	T/T	0.857	8.39 × 10^−5^	0.950	0.989
rs161334287	A/A	T/T	0.857	8.72 × 10^−5^	0.950	0.989
rs598937573	D/D ^2^	I/I	0.838	1.62 × 10^−4^	0.950	0.989
rs406431156	T/T	C/C	0.739	2.50 × 10^−2^	0.389	0.604

^1^ The insertion allele at the validated marker is AAT and the deletion allele is A. ^2^ The deletion allele is G and the insertion allele is GAAT.

## Data Availability

Publicly available datasets were analyzed in this study. This data can be found here https://www.ebi.ac.uk/ena/browser/view/PRJEB40528 (accessed on 1 February 2021) for ChIP-seq [41]. Data for CAGE can be found here https://www.ebi.ac.uk/ena/browser/view/PRJEB34864 [27] (accessed on 20 February 2021).

## Supplementary Materials

The following are available online at https://www.mdpi.com/article/10.3390/ani11071907/s1, supplementary Table S1: additional details on genotyping reagents; Table S2: Hardy-Weinberg Equilibrium analysis and animal counts by genotype for population 1; Table S3: Hardy-Weinberg Equilibrium analysis and animal counts by genotype for population 2; Table S4: Genotypes at each regulatory element marker tested that yielded a significant association (*p* < 0.05) with SRLV phenotype of resilience in sheep in population 2.
