# Supplementary material for: A DNA Regulatory Element Haplotype at Zinc Finger Genes Is Associated with Host Resilience to Small Ruminant Lentivirus in Two Sheep Populations
Source: Animals (Basel). 2021 Jun 26;11(7):1907. doi: 10.3390/ani11071907 (PMC8300134; doi:10.3390/ani11071907)
Supplement: Supplementary file 1 [file animals-11-01907-s001.zip › animals-1213484-SI.pdf]

Table S1. Supplementary table of variants tested and their corresponding genotyping reagents from the manufacturer and primer and probe sequences designed for this study. Probe 1 was labeled with VIC dye and probe 2 was labeled with FAM dye.

dbSNP identifier	Assay ID	Amplification Primer1	Amplification Primer 2	Probe 1	Probe 2
rs414403144	ANXG4UZ	TTGCTGCGAGTTAGCTACGA	AGCAAAAAGACATAACTGGGAATAAAAAGG	CACTCATCAATAATAAATAC	ACTCATCAATAGTAAATAC
rs399973518	ANYMYEX	CCTTTCCTGGCTACATACATGCAT	CCTGTAGAATGGACTACACAGCTTATAAATATTAA	TGAGGCATCGTTGTTAC	TAGTGAGGCATCTTTGTTAC
rs193645605	ANDKC49	TGAACAGTAAGTAGCTCTTTGGCAAT	GGGCAAAGGTTCATTTGTATGTGAA	AAGTGAGCCTTTTTCA	AGTGAGCCCTTTTCA
rs193645606	ANEP6P6	CAGAAATGAGGTTACCATGGAGCAT	GCTTTGGGTAGGACTAGGAGTTCT	TTGCTAAGGGCAAAGC	TTGCTAAGGACAAAGC
rs400257959	ANFVZA3	CTCACACCTGAATTAGCCTTTCCT	GTTGCCTTAAGTCAATGTCAGCAAT	TGGCTGCTACAGTGC	TGGCTGCTTCAGTGC
rs418789060	ANGZUVZ	CAGTGCGCTGTCTTTTTTCCT	ACATCAGAGAGGTACTTTATACATAGTT	CAACTATAAAGGAAAATAAAT	ACTATAAAGGACAATAAAT
rs161334222	ANH6NFX	TGCTGACATTGACTTAAGGCAACTAT	CCTCTTTTTTCTTGATACCTTTTCTTTGACA	CCTCTCTGATGTTGTTATC	CTCTCTGATGTCGTTATC
rs398476053	ANKCGZV	ATAACTATGTATAAAGTACCTCTCTGATGT	CTCCAGCCGTTTTCTTCTGTTTAAA	AAAAGAGGGATATTTGC	AAGAGGGCTATTTGC
rs414155747<br>rs425583788	ANMGCKT	TTGCTCTCATCTTTTAAACAGAAGAAAACG	GCTGAACATTCATGAAAGTTCCTTTCTTT	CCCCTCCGCCTCCAG	CCCCTCCAACTCCAG
rs426498685	ANNK46P	ATAAAGTTATCACAACAAAGCAGAGCTG	GCCCCTCCTCGCTTTTCTG	AGGGTCAAGAGAAAAGA	AGGGTCAAGAAAAAAGA
rs587824303	ANWDA2C	ATAAAGTTATCACAACAAAGCAGAGCTG	GCCCCTCCTCGCTTTTCTG	CTCCTCCATTCTCCGACTC	CTCCTCCATTCTCCACTC
rs416493683	ANYMX66	ATAAAGTTATCACAACAAAGCAGAGCTG	GCCCCTCCTCGCTTTTCTG	CGAAGCCCTATTTTT	CGAAGCCCTTTTTTT
rs427575002	ANZTTR3	GGGCTTCGCCAGAAAAGC	CACTGGAAATTCCCCTACAACGT	AACAGTCGCTGGAGCC	CAGTCGCAGGAGCC
rs406156873	AN2XMCZ	GGGAATTTCCAGTGCCAGTATGG	TTTTCCTTTCCCTCGTCCCTTTT	CCTCCCCAAAGGACAG	CTCCCCAAAAGACAG
rs417694680	AN33FWX	GCCAGTATGTCCTCCCCAAA	TTTTCCTTTCCCTCGTCCCTTTT	CCTTTCCCTCCTCCCTG	CCTTTCCCTTCTCCCTG
rs428884512	AN49AGV	GCCAGTATGTCCTCCCCAAA	TTTTCCTTTCCCTCGTCCCTTTT	CCTTTTCCTCCGACGCCT	CTTTTCCTCCAACGCCT
rs407355422	AHWSLF7	AAGTCTCACCCGGAAAACGAA	GGCATCAAAGGCCTCTTGGT	TCGTCAAAGGCCTCTTG	TCGTCAAAGCCCTCTTG
rs408781178	AHX1JMF	CTGAGCACACACAGACATAAACTTG	ACCTCAGAAAATTTATCTGTTTCTCAGATTTCT	AGGGCAGACATTATAG	AGGGCAGACGTTATAG
rs397514112*	AHN1R6J	CGAATGGATCTTCAAGGCTTA	CAGCTTTTCCATGCAGAGTC	TCCAATAAAATATGAC	TCCAATAAAATGACTT
rs599110985	AN9HXMP	TGCATGCCTAAAACGTAGGGAAAA	CTAGAAGATAGGGCAGCAAAGGA	ATTTATTGTTATCTTAAAACTTTT	ATTGTTATCTTAAAAATTTT
rs411076283	ANAANZF	TCTAGGTTTATCCAACTCCCTCTTGAG	GGCCAGTTCCTGACTCTTCTG	CAACTTAGCCACCTTCCCA	AACTTAGCCACTTTCCCA
rs413209326	ANCFHKD	GAGAACAGAACCGCCCTTGA	GCCGTTCCCCTTTAGCAATAATTTT	CAGACCCGCTTTCCA	CAGACCCACTTTCCA
rs193645607	ANDKC6E	GCAGTGCCCACCACACTA	GCTGGTTGCTTCTCTGACTACA	AAGAGCAAAGGGCCCTAC	AAGAGCAAAGGTCCCTAC
rs407841455	ANEP6RC	GGCCTCGCTTGCATCCT	GCAAAAATAAGCAAGGACGAAACCT	TGGTAGTCGTGGGTTC	TGGTAGTCTTGGGTTC
rs589283496	ANH6NF3	GTGTTCTGAATGCCAAATACTGCA	ACCCCTTTTTGAAGGTCTTGCT	AAGATTACGGACCGACCTG	AAGATTACGGACGACCTG
rs161334284	ANKCGZZ	GACCTGAGAGCAAGACCTTCAAAA	CAGTACACTGGTGTCTTGAGTTCTT	TGCAAAATCCCGTGGACTA	ATGCAAAATCCCTTGGACTA
rs161334287	ANMGCKX	CCAGTGTACTGGTTTGGACATACAA	GCTTCCGGAGGCTTTGCT	CCATAGAATGTATTCATACTTTGT	CCATAGAATGTATTCATACTTAGT
rs598937573	ANNK46V	CCGGAAGCCTGGATTCTGTTT	TGCTAGGTTTGGAAGTGACTTGAAA	CCCTTACAGAATAATGGAC	CCCTTACAGAATGGAC
rs420471584	ANRWUCP	CTTGTGAACTTCTGAAGACACGTTTT	GCTCCCAAACATCCCAAATTAGACT	CTAAAGGAAGAAAACATTCT	AAGGAAGAAAGCATTCT
rs161334474	AHPAKK0	GCCTCCTCACGTCTAGAAGTTAG	AAGAAGAGAGGTGCGAAAGCA	CGCAGGACCCTCC	CGCAGAACCCTCC
rs604883819	ANZTTYV	GGTGTGTAAAACAGCGAAAATGAGA	GAAGCGGAGAACCGTTTGAG	AGAAGAACATGACTTATTTCA	AAGAACATGACATTTCA
rs418383872	AN2XMJT	GCCACTTCCGGTAGCTAACG	CACCAGCCCTCGACTCC	ACCCGGGAGCTCC	CCCGCGAGCTCC
rs406431156	AN33F4P	AAATTGCTGATATTTGACTGTACAAAAATGGA	TGGAGATAAAGCCAGAACAATTTGCT	AATTCTCACACAAAAAG	CTCACGCAAAAAG
rs427605255	AN49APM	GCAGCAAGCACTGAGTAAACG	ACCCCACAACCACTGAATCCTA	TTTTCATTCCACAACCTGAG	TTCATTCCACCACCTGAG
rs161334705	ANAAN77	CTTCCGGGCCGCTCTAG	AAAAAAGCAAAGAAAACTTCAGCGA	TCCTCCACAGAGGCGA	CCTCCACCGAGGCGA
rs405931912	ANCFHT4	GCCGCCAGCGGAAGT	GTGTCCCCATGTGCAGACT	TGGCCGACACCCCCG	TGGCCGAGACCCCCG

*this is the validated marker and the assay was designed previously (White, 2014).

Table S2. Hardy-Weinberg Equilibrium analysis and animal counts by genotype for population 1 at each tested marker. Allele nucleotide bases are shown for SNPs: adenine (A), cytosine (C), guanine (G), and thymine (T), or insertion (I) and deletion (D) for small indels. * Previously validated marker.

Variant	Animals Genotyped	Genotype 1	Genotype 1 count	Genotype 2	Genotype 2 count	Genotype 3	Genotype 3 count	Chi-squared P-value
rs414403144	164	T/T	90	T/C	66	C/C	8	0.645
rs399973518	164	G/G	90	G/T	66	T/T	8	0.645
rs193645605	164	A/A	78	A/G	72	G/G	14	0.900
rs193645606	163	T/T	48	C/T	86	C/C	29	0.673
rs400257959	162	T/T	52	A/T	82	A/A	28	0.905
rs418789060	164	C/C	69	A/C	74	A/A	21	0.986
rs161334222	164	C/C	48	T/C	89	T/T	27	0.416
rs398476053	164	A/A	53	A/C	83	C/C	28	0.898
rs414155747& rs425583788	156	GC/GC	49	GC/TT	79	TT/TT	28	0.925
rs587824303	164	I/I	157	I/D	7	D/D	0	0.962
rs416493683	160	T/T	76	T/A	70	A/A	14	0.932
rs427575002	163	A/A	67	A/T	75	T/T	21	1.000
rs406156873	161	G/G	146	G/A	15	A/A	0	0.825
rs417694680	164	G/G	157	G/A	7	A/A	0	0.962
rs428884512	161	T/T	48	C/T	84	C/C	29	0.761
rs407355422	163	C/C	48	G/C	86	G/G	29	0.673
rs408781178	157	A/A	48	A/G	80	G/G	29	0.912
rs397514112*	163	I/I	48	I/D	87	D/D	28	0.564
rs599110985	164	T/T	48	G/T	87	G/G	29	0.627
rs411076283	163	T/T	48	C/T	86	C/C	29	0.673
rs413209326	163	T/T	48	C/T	86	C/C	29	0.673
rs162203030	127	G/G	37	A/G	65	A/A	25	0.934
rs193645607	164	G/G	122	G/T	40	T/T	2	0.815
rs407841455	164	G/G	48	G/T	87	T/T	29	0.627
rs589283496	163	I/I	48	I/D	79	D/D	36	0.949
rs161334284	164	G/G	48	G/T	87	T/T	29	0.627
rs161334287	163	A/A	48	A/T	86	T/T	29	0.673
rs598937573	163	D/D	48	I/D	87	I/I	28	0.564
rs420471584	160	C/C	66	T/C	80	T/T	14	0.328
rs161334474	164	G/G	67	G/A	82	A/A	15	0.359
rs161334479	163	AA	40	AG	91	GG	32	0.314
rs604883819	163	I/I	122	I/D	40	D/D	1	0.492
rs418383872	164	C/C	101	C/G	49	G/G	14	0.098
rs406431156	164	T/T	68	T/C	85	C/C	11	0.072
rs427605255	164	A/A	76	A/C	65	C/C	23	0.339
rs161334705	148	G/G	57	T/G	66	T/T	25	0.736
rs405931912	164	C/C	78	C/G	71	G/G	15	0.980

Table S3. Hardy-Weinberg Equilibrium analysis and animal counts by genotype for population 2 at each tested marker. Allele nucleotide bases are shown for SNPs: adenine (A), cytosine (C), guanine (G), and thymine (T), or insertion (I) and deletion (D) for small indels. * Previously validated marker.

Variant	Animals Genotyped	Genotype 1	Genotype 1 count	Genotype 2	Genotype 2 count	Genotype 3	Genotype 3 count	Chi-squared P-value
rs416493683	377	T/T	311	T/A	65	A/A	1	0.454
rs193645606	366	C/C	111	C/T	184	T/T	71	0.945
rs418789060	378	A/A	81	A/C	197	C/C	100	0.682
rs161334222	374	T/T	114	T/C	189	C/C	71	0.896
rs414155747& rs425583788	376	GC/GC	235	GC/TT	131	TT/TT	10	0.253
rs427575002	374	A/A	101	A/T	197	T/T	76	0.531
rs407355422	375	G/G	115	G/C	189	C/C	71	0.913
rs397514112*	379	I/I	72	I/D	193	D/D	114	0.832
rs406431156	377	T/T	104	T/C	192	C/C	81	0.910
rs399973518	376	G/G	154	G/T	184	T/T	38	0.285
rs193645605	377	A/A	312	A/G	64	G/G	1	0.476
rs599110985	377	G/G	115	G/T	190	T/T	72	0.918
rs411076283	376	C/C	115	C/T	190	T/T	71	0.892
rs193645607	376	G/G	219	G/T	132	T/T	25	0.705
rs407841455	371	G/G	71	G/T	186	T/T	114	0.952
rs161334287	380	A/A	72	A/T	191	T/T	117	0.930
rs598937573	376	I/I	115	I/D	189	D/D	72	0.937
rs420471584	375	T/T	74	T/C	197	C/C	104	0.539
rs604883819	378	I/I	351	I/D	26	D/D	1	0.786
rs398476053	374	A/A	236	A/C	128	C/C	10	0.319

Table S4. Genotypes at each regulatory element marker tested that yielded a significant association (p <0.05) with SRLV phenotype of resilience in sheep in Population 2 that were tested in both populations. The most extreme genotypes associated with resiliency and susceptibility to SRLV and log-transformed adjusted mean proviral concentration difference (Genotypic Log_{10} Conc. Diff.) between genotypes for Population 2 (crossbred Rambouillet, Columbia) is shown. The p-value calculated for the association analysis in population 1 and linkage disequilibrium (r^2) between the newly tested markers and the previously validated marker is also shown. Genotype allele nucleotide bases are shown for SNPs: adenine (A), cytosine (C), guanine (G), and thymine (T), or insertion (I) and deletion (D) for small indels.

Marker	Resilient Genotype	Susceptible Genotype	Genotypic Log_{10} Conc. Diff.	*P*-value	LD with validated marker* (r^2)
**rs193645606**	T/T	C/C	0.488	3.01×10^{-4}	0.989
**rs418789060**	C/C	A/A	0.396	3.49×10^{-3}	0.705
**rs161334222**	C/C	T/T	0.487	2.88×10^{-4}	0.989
**rs398476053**	C/C	A/A	0.590	2.37×10^{-2}	0.312
**rs414155747& rs425583788**	TT/TT	GC/GC	0.580	3.38×10^{-2}	0.313
**rs427575002**	A/A	T/T	0.368	8.67×10^{-3}	0.678
**rs407355422**	C/C	G/G	0.486	2.75×10^{-4}	0.989
**rs397514112***	I/I[1]	D/D	0.479	2.77×10^{-4}	-
**rs599110985**	T/T	G/G	0.486	2.37×10^{-4}	0.989
**rs411076283**	T/T	C/C	0.487	2.78×10^{-4}	0.989
**rs407841455**	G/G	T/T	0.485	3.50×10^{-4}	0.989
**rs161334287**	A/A	T/T	0.479	2.71×10^{-4}	0.989
**rs598937573**	D/D[2]	I/I	0.483	2.90×10^{-4}	0.989
**rs406431156**	T/T	C/C	0.337	2.16×10^{-2}	0.604

*previously validated marker

[1] The insertion allele at the validated marker is AAT and the deletion allele is A.

[2] The deletion allele is G and the insertion allele is GAAT.
